# On the Prevention of Typhoid and Malarial Fevers*At a stated meeting of Madison County Medical Society held on the 10th day of May, 1904, a resolution was adopted that authorized the President to appoint a committee of three to prepare a paper on the prevention of Typhoid and Malarial Fevers, for popular information. The President appointed as the committee Drs. J. E. Morris, Sr., Lawrence Corley and J. D. Jordan.

**Published:** 1904-07

**Authors:** 


### On the Prevention of Typhoid and Malarial Fevers.*

Every individual knows that food, clothing and houses are not provided for him by nature. If he wishes to enjoy the comforts of life, he must exert his own energies to obtain them, or some one must do it for him. He recognizes the necessity of providing himself and those dependent upon him against want; but strange to say, he does not always appreciate the equally imperative duty of guarding himself and family against disease. He stirs himself to protect his growing crops against breachy stock and his tender flock against thieves and ravaging wolves, but when the invisible death-dealing microbe enters his home and devours the life-blood of his dearest ones, he sits in Turkish supineness and moans, or looks often in vain to drug stores and doctors for relief from the results of his own negligence.

The greater part of the sickness that prevails in this section can be prevented. Surprising as this assertion may appear to some, its truth can be demonstrated.

Malarial diseases prevailing here throughout the year, but more especially during the summer and fall, as chills and fever, remittent or bilious fever, congestive or pernicious fever, black jaundice or black water fever, not only causes great suffering, loss of time, money, health and life directly, but predisposes the afflicted persons to neuralgia, rheumatism, digestive disturbances, kidney troubles, consumption and a host of other formidable maladies. Could the loss in time, money and life occurring in Madison county every year from malarial affections be accurately computed, the results would be startling. No further evidence would be needed to convince the most careless of the urgent necessity of using all possible preventive measures.

Can malarial affections be prevented in Madison county?

We most emphaticlly say, yes.

How?

Malarial affections are caused by a living minute parasite called by the doctors hæmosporidia or plasmodium malaria. The parasite

*At a stated meeting of Madison County Medical Society held on the 10th day of May, 1904, a resolution was adopted that authorized the President to appoint a committee of three to prepare a paper on the prevention of Typhoid and Malarial Fevers, for popular information. The President appointed as the committee Drs. J. E. Morris, Sr., Lawrence Corley and J. D. Jordan.

can not be seen by the unaided eye. A microscope of large power is required to bring them into view. Three different varieties of the parasite have been recognized producing different forms of malarial fever. Sometimes more than one variety may exist in the blood of the same patient causing an irregular continuous form of fever. The parasite enters into the red cells of the blood and feeds upon the corpuscle. A minute description of the germ and its effect upon the body would probably prove more puzzling than instructive. Sufficient to say that all malarial affections with their disastrous consequences depend upon the introduction of the parasite into the blood. Potatoes, however favorable the soil, the season and the cultivation, can not be grown without the potato seed is first placed in the soil; likewise no malarial disease can originate until the malarial parasite has been first introduced into the blood. No seed, no potatoes; no parasite, no malaria. For many centuries the origin of the disease remained a profound mystery to the wisest and best-informed physicians. Fact after fact relating to the affections were observed and recorded, here a little and there a little, until finally, in 1880, Dr. Laveran, an Italian physician, discovered the parsite. His discovery was soon confirmed by observations made in nearly all portions of the civilized world. Still there remained the hard problem, how does the germ get into the blood?

It was known that the parasite being a heavy body could not float in the air, and yet it was evident that the disease came through the air. Experiment proved that the parasites could not enter the blood by way of the stomach. Water and food containing the germs were harmless when swallowed. Additional observations made it clear that some insect must be the medium of conveyance. Finally it was demonstrated that the mosquito was the intermediate host through which the disease was propagated. Not all mosquitoes are alike guilty, but one particular species known as Anopheles, and of the species the Anopheles claviger are the most dangerous. The mystery that so baffled the patient investigation of physicians was now cleared up. It is known that the malarial bearing mosquito is active only at night. Hence persons may sleep with perfect immunity in the thoroughly infected swamps if protected from sundown to sunrise by mosquito-bars. This fact has been proved by a large number of experiments, two of which will now be related: In that dangerous region, the Campania (near Rome, Italy), Dr. Sanbron and a friend spent the nights from June to September,

1900, in a well-screened hut and escaped infection. A battalion of Japanese troops in Formosa were protected from mosquitoes for 161 days during the malarious season and entirely escaped the disease. An unprotected battalion in the same place during that time developed 259 cases of malaria. The efficacy of the simple precaution of screening has become so well known that it is employed on a large scale in many malarial regions. A son of Dr. Manson, a healthy man who had never had malaria, nor been in a malarious region, allowed himself to be bitten in England by infected mosquitoes brought from Italy. In a few days he came down with a sharp attack of malaria, and plasmodia (that is the malarial parasite) which were not present in the blood before were found in abundance. If space permitted, many other incidents could be related demonstrating beyond a question of doubt, that the mosquito does introduce the malarial parasite into human blood. In all cases where the infected mosquitoes have been promptly examined the sporozoids and crescents of the malarial parasite have been found. It has not been proven that malarial diseases are ever communicated to human beings in any other way than through infected mosquitoes. All known facts warranted the conclusion that the mosquitoes are the only medium through which the malaria parasites are transmitted. Prevention of malarial diseases then consists in protection from the bites of infected mosquitoes. (Although it has been proven that taking from two and one-half to five grains of quinine three times a day for four days in the week during the sickly season affords marked protection.) Obviously the better method of protection consists in a destructive war upon the pestiferous insects. When it is remembered that the great scourge, the yellow fever that annually threatens Texas is communicated through mosquitoes, though of a different species, another most cogent reason is added for exterminating the pest. In carrying on this warfare of extermination some uniform plan of action should be adopted.

The following extract bearing on this subject is quoted from an address delivered at Austin, Texas, on the 30th of March, 1904, to a conference of local county physicians by the State Health Officer, Dr. George R. Tabor:

"The United States Department of Agriculture last year issued a bulletin on the destruction of mosquitoes, which I reproduce, with a few changes, which should be placed in the hands of every person in Texas for their examination:

"You are responsible for the mosquitoes in your own house and dooryard. Read the rules carefully:

"1. Mosquitoes breed only in water; usually fresh, standing water in artificial places.

"2. Mosquitoes occur in the vicinity in which they breed. Invasions from long distance are exceptional.

"3. The young mosquito or 'wiggler' lives in water at least ten or twelve days.

"4. Although the wigglers live in water, they must come frequently to the surface to breathe.

"5. Coal oil on the surface of water prevents the wigglers from breathing.

"6. Destroy the breeding places and you will destroy the mosquito.

"7. Empty the water from all tubs, buckets, cans, flower pots, vases once every forty-eight hours.

"8. Fill or drain all pools, ditches and various excavations, as post-holes left unfilled, etc.

"9. Change regularly every day all water needed in chicken coops, kennels, etc.

"10. Treat with coal oil all standing water which can not be screened or drained (one ounce of oil will cover fifteen square feet of surface). The oil does not affect the water for use if the water is drawn from below.

"11. Put wire netting over cisterns, wells and tanks of water in everyday use.

"12. Places in which it is undesirable to place oil, such as watering troughs for stock, lily ponds, etc., can be kept free from wigglers by putting in gold fish. The nymphs of dragon flies and tadpoles of frogs also feed on the wigglers.

"13. Inspect all cesspools and see that the covers are absolutely tight.

"14. Clean away all weeds, grass and bushes about ditches, ponds and other possible breeding places, since these afford a hiding place for the adult mosquitoes.

"15. Clean up vacant lots and back yards of all cans, tins, bottles and rubbish.

"16. First do away or treat all places where mosquitoes are known to breed, and then begin work on places where they might breed.

"17. As a citizen of your community you should feel a personal responsibility for the destruction of the mosquitoes in your district, and seek to cooperate with your neighbors in the work of doing away with breeding places. Inspect and treat with coal oil gutters, culverts, ditches, manholes, catching basins, etc., along the road side.

"18. Where oil is applied to standing water it must be distributed evenly over the surface. Use a hand syringe, or, if the area is great, a knapsack sprayer.

"19. Houses should be cleared of all winged mosquitoes by the burning of insect powder. The mosquitoes will fall to the floor, and should be collected and burned.

"20. Relief in any community or district depends entirely upon the cooperation of the members of the community.

"I want to emphasize the following suggestions requesting you to bend your efforts to their adoption immediately upon your return to your home. Have your city council enact an ordinance prohibiting the use of water barrels or prescribe penalties for persons using them without screening and oiling the barrels. Make it a penalty for any person to use barrels or other vessels for water who permit mosquitoes to breed in them.

"Have your city council enact an ordinance requiring all old unused cisterns and wells to be filled. The unused cisterns harbor mosquitoes all winter and are the greatest of all breeding places for them in summer. Cisterns in use should be screened always and treated to oil as suggested before, but if you find objection urged to the use of oil suggest that minnows or small fish be placed in them. They will feed on the wiggletails and eggs and prevent the production of mosquitoes. But under all circumstances use the extra precaution of screening the cisterns. Don't overlook the elevated cisterns and water tanks along the railways and around cotton gins and mills. Require those to be screened and oiled. Ordinary ponds and pools which never go dry and are large enough to contain fish are not breeding places for mosquitoes, as the fish destroy the larvæ, but all weeds, grass and brush should be kept cut away. Such ponds, however, should not be permitted in a progressive city or town which is supplied with waterworks. All other places containing water should be drained or filled. If drainage or filling is not possible they should be kept oiled twice weekly.

"Where the mosquitoes already exist (in residences) they may be destroyed by fumigation of houses. All the rooms should be tightly closed, paste paper over the cracks and openings, burn two pounds of sulphur to one thousand cubic feet of space; keep the room closed eight hours."

Now a few words may be added in regard to the prevention of the spread of typhoid fever, often called slow fever. Typhoid fever is caused by a living germ or bacteria called typhosus bacillus. Nothing else but this particular germ causes this fever. No wheat can be raised unless the wheat grains have first been placed in suitable soil; likewise, it is utterly impossible that typhoid fever can be developed without the typhoid bacilli have been first introduced into the human system.

How do typhoid germs get into the system? Mainly through the mouth. The bacilli are exceedingly small. They are made visible to the human eye only through a powerful microscope. Nevertheless they exist as much so as thorns, thistles and crab grass. They are very tenacious, clinging closely to fingers, hands, spoons, clothing and every object in which they come in contact; hence the difficulty of removing them by a simple washing. Outside of a living body the typhoid germs may live varying lengths of time according to the condition of the medium in which they are placed. Sunlight, dryness, and a temperature less than a boiling point of water and chemical solutions prove rapidly fatal to them. Freezing does not destroy them. Dark, damp, ill-ventilated, filthy recesses form favorable conditions for their speedy development. Flies carry the microbes from privy vaults and other places where infected material has been deposited, to the hands, faces, dining and living rooms of the people from where they may easily be transferred to the mouths of susceptible persons.

It is known that typhoid germs may retain their vitality in milk and in drinking water many weeks and in the soil for many months. *A well once infected may be dangerous for years.* The common mode of conveyance of the germs to the mouth is through drinking water, milk or cold foods or through infected hands, spoons, or other utensils. Walking cases of typhoid, by depositing the germs with the evacuations from the bowels in different places, sometimes cause a reproduction of the disease in new localities.

It is often difficult and frequently impossible to trace the source of the infection in the first case occurring in a community. When the first case develops, the interest of the whole community is deeply concerned in preventing the development of other cases. The following measures to prevent the spread of typhiod fever are suggested:

1. Isolate or quarantine the patient.

2. If it is suspected that the drinking water is contaminated, boil all the water used from the suspected source.

3. Keep all privies, commodes, etc., thoroughly disinfected at all times. Disinfect all discharges from patient by strong chemical solutions prepared for that purpose.

4. Thermometers, syringes, tubes, spoons, dishes, other utensils, clothing, bedding, towels, handkerchiefs and everything brought into close contact with the patient should be disinfected by boiling or by chemical solutions prepared for the purpose.

5. The hands of nurses and attendants should be frequently washed in a disinfectant solution.

Scrupulously studied cleanliness in all things should be carefully maintained throughout the disease and for several weeks after the case terminates. By no means permit the discharges from the patients or waste from the sick room to be carelessly disposed of. Remember the health of the whole community, including some lives, may depend upon the manner of carrying out methods of prevention.

In adopting measures to prevent sickness each individual family may do something, but a whole community cooperating will accomplish a great deal more. The statutes of Texas provide that the commissioners court may establish health districts and appoint a board of health under the direction of the State Health Officer in unincorporated towns and villages.

Now will you, a public spirited citizen, continue to remain in supine inactivity until one or more members of your household have been racked by burning fevers, or it may be carried to their long home by a company of sympathetic friends?

Will the grave only move you? When sickness or death enters your door ask yourself the question, "What have I done to prevent it?" How far are you as an individual or as a member of the community responsible for the occurrance of sickness or death that could have been prevented? These self-interrogatories are not intended for the family on the creek or your neighbor over the way only, but for you! you!

An ounce of prevention is worth a ton of cure.

J. D. Jordan,
Lawrence Corley,
Jno. E. Morris,
Committee.

—*In Madison Meteor, June 3, 1904.*

